# Effects of 8-week alkaline diet and aerobic exercise on body composition, aerobic performance, and lipid profiles in sedentary women

**DOI:** 10.3389/fnut.2023.1339874

**Published:** 2024-01-04

**Authors:** Nehir Yalcinkaya, Ozkan Isik, Malik Beyleroglu, Dogan Erdogdu, Guner Cicek, Dario Novak

**Affiliations:** ^1^Faculty of Sports Sciences, Sakarya University of Applied Sciences, Sakarya, Türkiye; ^2^Faculty of Sports Sciences, Balıkesir University, Balikesir, Türkiye; ^3^Directorate of Sports Sciences Application and Research Center, Balikesir University, Balikesir, Türkiye; ^4^Sakarya University, Sakarya, Türkiye; ^5^Faculty of Sports Sciences, Hitit University, Çorum, Türkiye; ^6^University of Zagreb Faculty of Kinesiology, Zagreb, Croatia

**Keywords:** low-density lipoprotein, high-density lipoprotein, nutrition, sedentary lifestyle, sedentary women

## Abstract

**Background:**

Diet composition can affect systemic pH and acid–base regulation, which may in turn influence exercise performance.

**Purpose:**

It was aimed to determine the effects of the alkaline diet and 8 weeks of aerobic exercises on body composition, aerobic performance, and blood lipid profiles in sedentary women.

**Methods:**

Thirty-two sedentary women participated in the study voluntarily. The research was designed with a true-experimental design and the participants were divided into four different groups as the control group, aerobic exercise group, alkaline diet group, and alkaline diet + aerobic exercise group. The body compositions, aerobic exercise performances, and lipid profiles of sedentary women were measured as pre-test and post-test. In the analysis of the obtained data, One-Way ANOVA with Bonferroni *post hoc* test was used.

**Results:**

It was observed that the alkaline diet consumed with 8 weeks of aerobic exercises caused a 5.17% decrease in BMI and an increase of 42.07 and 37.62% in VO_2max_ and aerobic test durations, respectively (*p* < 0.05). In addition, when lipid profiles were examined, it was determined that there was no statistically significant difference in HDL-C levels (*p* > 0.05). Despite that, there were statistically significant differences in TG and LDL-C levels (*p* < 0.05). According to this result, it was determined that there was a decrease in TG and LDL-C levels by 37.61 and 20.24%, respectively.

**Conclusion:**

An alkaline diet consumed with 8 weeks of aerobic exercises in sedentary women has positive effects on improving body composition, aerobic exercise performances, and TG and LDL-C levels.

## 1 Introduction

Dietary intervention is a cornerstone of weight loss treatment. Most recommended dietary regimens for weight loss focus on energy content and macronutrient composition. In order to achieve weight loss, energy intake must be less than energy expenditure (1). The dietary composition can be modified to reduce acid loads and improve acid–base balance (2) and new diets and supplements have emerged to fight against acidosis (3, 4). Maintaining acid–base homeostasis is vital for metabolic processes and strongly influences the body’s acid–base balance on nutrition, human physiology, and health. Dietary acid load is an important determinant of systemic pH, acid–base regulation, and metabolism (5). Protein-rich foods, including meats, fish, eggs, grains, and dairy products, are acidogenic as they increase acid production in the body, and the metabolism of sulfur-containing amino acids (cysteine and methionine) by the liver results in the production of hydrogen ions, which can lower blood pH (6, 7).

Alkalizing diets are characterized by a negative potential renal acid load (also called low-PRAL) diets and low-PRAL diets generally consist of all vegetables and fruits, alkali-rich, and low-phosphorus beverages. Fruits and vegetables contain potassium, which releases the precursors of bases in the bloodstream, thereby creating an alkalizing effect (3, 7). Dietary acid–base load is a balance between acidogenic foods (foods containing protein) and alkalinogenic foods (fruits and vegetables) that provide base precursors (6). Alkaline supplementation can overcome the acidosis caused by hunger, thus leading to greater weight loss and better running performance (8). Dietary acid load, measured as the PRAL of the diet, influences systemic pH and acid–base regulation (9). A low-PRAL-based diet has many advantages, such as improving cardiovascular health, lowering blood pressure and body weight, lowering blood lipids such as cholesterol, and protecting the body from diseases (10).

Physical exercise causes metabolic changes that affect acid–base balance in skeletal muscles and other tissues. Acid–base balance is essential for maintaining health and physical performance indicators. While organic acids are produced in the body during basal metabolism, physical exercise can lead to additional acid production in the body (11). The development of acidosis during an intense exercise, traditionally increased lactic acid production, causes the release of a proton and the acid salt is explained by causing the creation of sodium lactate. Based on this explanation, if the lactate production rate is high enough, the cellular proton buffering capacity can be exceeded, and this situation can cause a drop in cellular pH (12). Diet type and exercise strongly affect the body’s acid–base balance through their own mechanisms. However, they can also interact, affecting exercise performance. It has been hypothesized that creating a more alkaline systemic environment by reducing dietary acid load may increase the clearance of protons and inhibitory molecules affecting working muscles during exercise-induced acidosis, thereby improving aerobic and anaerobic exercise performance (13).

In some studies, it is known that increased hydrogen (H^+^) concentrations in the muscles and blood during high-intensity exercise cause acidosis, which is considered one of the causes of fatigue (12, 14), and the acid–base state has the potential to affect physical performance before exercise (15, 16). Recent studies suggest that ingestion of a particular diet can alter blood buffering capacity and that changes in blood buffering capacity may affect low- or high-intensity aerobic and anaerobic exercise performance (17, 18). When the literature was examined, there were limited studies in which a low-PRAL diet is consumed with exercise (19–21). In addition, these studies are mostly cross-sectional and longitudinal studies are almost nonexistent. In this context, this study aimed to determine the effect of 8 weeks of aerobic exercises and a low-PRAL diet on body composition, aerobic exercise performance, and lipid profile of sedentary women. So, it was hypothesized that 8 weeks of aerobic exercise and a low-PRAL diet would affect the body composition, aerobic exercise performance, and lipid profile of sedentary women.

## 2 Methods

### 2.1 Calculation of the sample size

In order to generalize the research results, a power analysis was performed to determine the sample groups. The total number required to find the expectation of a medium effect size (*f* = 0.40) to be statistically significant in revealing the effect of the low-PRAL diet and aerobic exercise was determined as 32 (eight participants for each group; α = 0.05; 1−β = 0.95).

### 2.2 Participants

Thirty-two sedentary women (X̄_age_ = 48.12 ± 5.18 years) voluntarily participated in the study. All participants were medically checked before starting the study and were non-menopausal women. Participants were selected as moderately healthy, sedentary women who did not use any chronic medication in the last 6 months, and did not experience any skeletal-muscular system injuries. In addition, the participants had not followed a special diet program or any nutritional supplement in the last 6 months. Detailed information about the purpose and content of the study was given to the participants and an informed consent form was signed. Ethical approval of this research was obtained from Sakarya University of Applied Sciences (Protocol No: 26428519/044/14).

### 2.3 Experimental design

The research was designed with a true-experimental design and the participants were divided into four different groups as the control, aerobic exercise, low-PRAL diet, and low-PRAL diet + aerobic exercise group. At the beginning of the study, the participants’ body compositions (BMI, fat mass %, and fat-free mass %) were analyzed by using a bioelectrical impedance analyzer. The High-Density Lipoprotein-Cholesterol (HDL-C), Low-Density Lipoprotein-Cholesterol (LDL-C), and Triglyceride (TG) were analyzed as lipid profiles from blood samples. In addition, aerobic exercise performances (Aerobic Test Duration and VO_2max_) analyzed through the Bruce Treadmill test were analyzed. After the pre-test measurements, the participants in the experimental groups applied a total of 24 sessions of exercise 3 days a week (Monday, Wednesday, and Friday) for 8 weeks. During pre- and post-test measurements, the diet groups consumed a low-PRAL diet and the control group consumed a standard diet and it was kept under control that they did not participate in physical activity programs. During the test period, participants were warned not to consume beverages such as alcohol and energy drinks. In addition, they were asked to get a good sleep the night before the measurements. Post-test measurements were taken at the same time of day as pre-test measurements, 48 h after the last exercise.

#### 2.3.1 Measurement of body composition

The heights of the participants were measured with a portable Wall Mounted Height Meter (Mesilife Sw-G06b) measuring with ±1 mm precision. Body mass index, fat mass, and free-fat mass ratios of the participants were measured in sedentary mode with TANITA BC-418 (Tanita Corp., Tokyo, Japan) brand bioelectrical impedance analyzer in the morning on an empty stomach, wearing shorts and a t-shirt.

#### 2.3.2 Exercise intervention and measurement of aerobic performance

Exercise groups applied 60-min walking exercises on the track and field track of the Sakarya University of Applied Science for 3 days a week for 8 weeks. The participants were included in the walking exercise for 1 h for each session after they had their breakfast. The participants applied walking exercises for 1 h at a speed of 4 km/h in the first and second weeks, 4.5 km/h in the third and fourth weeks, 5 km/h in the fifth and sixth weeks, and 6 km/h in the seventh and eighth weeks. Participants applied warm-up exercises for 10–15 min before starting the exercise and cool-down exercises for 5–10 min at the end of the exercise. Control and low-PRAL diet groups did not participate in any exercise program for 8 weeks, they were kept under control and recorded daily.

Sub-maximal Bruce treadmill test was used to determine the aerobic exercise performances of the sedentary women. Because the participants were sedentary and had a high average age a sub-maximal test was preferred. The test was performed on the treadmill in 3-min steps up to 85% of the participants’ estimated age-based Maximum Heart Rate (HR_Max_). Sub-maximal Bruce treadmill test starts at 2.7 km/h with a 10% incline after a general warm-up and increases speed and slope every 3 min. Bruce’s treadmill test was finished when the heart rate of the participants reached 85% of the estimated HR_Max_. The duration of the test was recorded and the estimated VO_2max_ was calculated by a formula using the test ending time (22). The sub-maximal heart rate (85% HR_Max_) of the participants was calculated using the formula [208 − (0.7 × Age) × 85%] (21). In the calculation of estimated VO_2Max_ (For women: Estimated VO_2Max_ = 4.38 × (Test duration) − 3.90) formula was used (23).

#### 2.3.3 Dietary intervention

The diets of the participants were specially prepared by a specialist dietitian. The daily calorie, fluid, and PRAL values that the participants would consume in their nutrition programs were calculated and low-PRAL diets were adjusted to 0 to −10 mEq/day by sedentary women. The diets of the experimental and control groups were calculated with the help of PRAL. PRAL is a value that reports the acid–base status of the foods we take orally after they pass into the stomach (24). The protein content of the PRAL formula is based on Cl^−^, PO_4_^3−^, SO_4_^2−^, Na^+^, K^+^, Ca^2+^, and Mg^2+^ (25). The PRAL value of foods (mEq/100 g) is calculated as: 0.49 × protein (g/100 g) + 0.037 × phosphorus (mg/100 g) − 0.021 × potassium (mg/100 g) − 0.026 × magnesium (mg/100 g) − 0.013 × calcium (mg/100 g) (2).

A German PRAL list published by the Institute for Prevention and Nutrition, Ismaning, Germany, was distributed to the participants (26). In the PRAL list, participants are advised on how to make food alkaline or acidic. The participants in the low-PRAL diet group consumed animal proteins like fish, chicken, and turkey meat for 8 weeks in their main menu only 3 days a week (exercise days). They consumed animal proteins for breakfast 7 days a week as curd cheese and powdered egg white. For 8 weeks, animal proteins were reduced, and mostly vegetable proteins (soybeans, lentils, beans, peas, chickpeas, etc.) were emphasized. The low-PRAL diet group, which is considered to be acidic drinks during the research, was not allowed to consume coffee, cola, milk, instant juices, and black tea for 8 weeks, but herbal teas were allowed (green tea, white tea, fennel, mint-lemon tea, and rosehip). As a result, individuals in the experimental group kept animal proteins limited for 8 weeks (fish, chicken and turkey meat, curd cheese, and egg whites), mostly vegetable-fruit, starchy vegetables (sweet potatoes), vegetable oils (almonds, avocados, walnuts, and nuts), and dried fruits (black raisins, dried apricots, dried figs, and dried dates). Animal proteins were included in the nutrition programs of the participants in the control group every day of the week for 8 weeks [only red meat, eggs, milk, yogurt, and cheese (cheddar, cream, and parmesan)]. In addition to consuming grains (oats, bread, pasta, and rice), they were also allowed to drink black tea, coffee, milk, acidic beverages (cola, etc.), and instant fruit juices. High-sugar fruits and dried fruits were also included, and 8-week diet programs were completed.

#### 2.3.4 Determination of lipid profile

In order to determine the lipid profiles of the participants, 5 cc blood samples were taken from the antecubital veins of the forearm between 08.00 and 09.00 in the morning for both pre-test and post-test measurements by specialist health personnel. Blood samples were collected in the physiology laboratory of Sakarya University of Applied Sciences, Faculty of Sport Sciences. Blood samples were centrifuged at 4,000 rpm. HDL-C, LDL-C, and TG were evaluated in the obtained plasmas.

### 2.4 Statistical analysis

IBM SPSS Statistics 24 software was used for the statistical analysis. One-way ANOVA with the delta approach was used to analyze the obtained data. Bonferroni *post hoc* test was preferred to determine the source of the difference between the groups. The percentage changes of the measured variables were calculated with the formula “% Δ = (Pretest − Posttest)/Pretest *100.” The confidence interval was chosen as 95% and values below *p* < 0.05 were considered statistically significant.

## 3 Results

When the percentage change in the body composition of the sedentary women was examined, it was determined that there was no statistically significant difference between the groups in terms of fat mass and free fat mass ratios (*p* > 0.05), but there was a statistically significant difference in BMI of sedentary women (*p* < 0.05). According to this result, there was a statistically significant difference between a low-PRAL diet consumed with 8 weeks of regular aerobic exercise with the BMI levels of the low-PRAL and Control groups. However, there was no statistical difference between the BMI levels of the aerobic exercise group and the aerobic exercise + low-PRAL diet groups. With 8 weeks of regular aerobic exercise, BMI decreased by 3.34%, with 8 weeks of alkaline diet consumption, by 1.26%, and a low-PRAL diet consumed with 8 weeks of regular aerobic exercise decreased the BMI of sedentary women by 5.17% (Table 1).

**Table 1:** Percentage changes of 8-week of alkaline diet and aerobic exercises on body composition.

Variables	Groups	*N*	X—± S.D.	*F*	*p*
%ΔBMI (kg/m^2^)	Aerobic exercise + Low-PRAL	8	−5.17 ± 2.4^a^	4.773	**0.008** ^*^
	Aerobic exercise	8	−3.34 ± 2.36^ab^		
	Low-PRAL	8	−1.26 ± 1.23^b^		
	Control	8	1.38 ± 1.85^c^		
%ΔFat mass (%)	Aerobic exercise + Low-PRAL	8	0.33 ± 4.47	1.518	0.232
	Aerobic exercise	8	2.09 ± 3.24		
	Low-PRAL	8	4.13 ± 4.94		
	Control	8	4.3 ± 4.42		
%ΔFree-fat mass (%)	Aerobic exercise + Low-PRAL	8	−0.03 ± 2.51	1.494	0.238
	Aerobic exercise	8	−1.41 ± 1.97		
	Low-PRAL	8	−2.06 ± 2.36		
	Control	8	−2.27 ± 2.46		

^*^p < 0.05; ANOVA; ^a,b,c^Different letter shows statistically difference between groups with the Bonferroni test.

When the percentage change in the aerobic performance of the sedentary women was examined, it was determined that there was a statistically significant difference in aerobic test duration and VO_2Max_ of sedentary women (*p* < 0.05). According to this result, there was a statistically significant difference between a low-PRAL diet consumed with 8 weeks of regular aerobic exercise with the aerobic exercise duration of the aerobic exercise, low-PRAL, and control groups. With 8 weeks of regular aerobic exercise, aerobic exercise duration increased by 11.44%, with 8 weeks of low-PRAL diet consumption increased by 11.66%, and a low-PRAL diet consumed with 8 weeks of regular aerobic exercise increased the aerobic exercise duration of sedentary women by 37.62%. Moreover, there was a statistically significant difference between a low-PRAL diet consumed with 8 weeks of regular aerobic exercise with the VO_2Max_ of the aerobic exercise, low-PRAL, and control groups. With 8 weeks of regular aerobic exercise, VO_2Max_ increased by 12.75%, with 8 weeks of low-PRAL consumption by 13.06%, and a low-PRAL diet consumed with 8 weeks of regular aerobic exercise increased the VO_2Max_ of sedentary women by 42.07% (Table 2).

**Table 2:** Percentage changes of 8-week of alkaline diet and aerobic exercises on aerobic performance.

Variables	Groups	*N*	X—± S.D.	*F*	*p*
%ΔAerobic test duration (min)	Aerobic exercise +Low-PRAL	8	37.62 ± 5.12^a^	55.549	**0.001** ^*^
	Aerobic exercise	8	11.44 ± 3.00^b^		
	Low-PRAL	8	11.66 ± 7.47^b^		
	Control	8	−5.28 ± 9.47^c^		
%ΔVO_2Max_ (mL/kg/min)	Aerobic exercise + Low-PRAL	8	42.07 ± 6.06^a^	55.923	**0.001** ^*^
	Aerobic exercise	8	12.75 ± 3.30^b^		
	Low-PRAL	8	13.06 ± 8.33^b^		
	Control	8	−5.37 ± 10.16^c^		

^*^p < 0.05; ANOVA; ^a,b,c^Different letter shows statistically difference between groups with the Bonferroni test.

When the percentage change in the lipid profile of the sedentary women was examined, it was determined that there was no statistically significant difference between the groups in terms of HDL-C (*p* > 0.05), but there was a statistically significant difference in TG and LDL-C of sedentary women (*p* < 0.05). According to these results, it was determined that a low-PRAL diet consumed with 8 weeks of regular aerobic exercise decreased the TG and LDL-C of sedentary women by 37.61 and 20.24%, respectively at a higher than the aerobic exercise and a low-PRAL diet groups (Table 3).

**Table 3:** Percentage changes of eight-week of alkaline diet and aerobic exercises on lipid profile.

Variables	Groups	*N*	X—± S.D.	*F*	*p*
%ΔTG (mg/dL)	Aerobic exercise + Low-PRAL	8	−37.61 ± 21.02^a^	3.779	**0.021** ^*^
	Aerobic exercise	8	−24.75 ± 25.63^ab^		
	Low-PRAL	8	−0.55 ± 28.72^b^		
	Control	8	−9.26 ± 19.12^ab^		
%ΔHDL-C (mg/dL)	Aerobic exercise + Low-PRAL	8	22.78 ± 63.43	0.989	0.412
	Aerobic exercise	8	4.88 ± 12.95		
	Low-PRAL	8	−5.73 ± 7.52		
	Control	8	10.59 ± 17.84		
%ΔLDL-C (mg/dL)	Aerobic exercise + Low-PRAL	8	−20.24 ± 16.79^a^	7.001	**0.001** ^*^
	Aerobic exercise	8	9.74 ± 18.33^b^		
	Low-PRAL	8	−4.03 ± 14.88^ab^		
	Control	8	10.91 ± 11.41^b^		

^*^p < 0.05; ANOVA; ^a,b,c^Different letter shows statistically difference between groups with the Bonferroni test.

## 4 Discussion

This study was carried out to determine the effect of a low-PRAL diet and aerobic exercise for 8 weeks on body composition, aerobic performance, and lipid profiles in sedentary women. In the current study, it was observed that there was no statistically significant difference between the groups in terms of fat mass and free fat mass ratios of sedentary women. However, there was a statistically significant difference in the BMI of sedentary women. According to this result, with 8 weeks of regular aerobic exercise, BMI decreased by 3.34%, with 8 weeks of alkaline diet consumption, by 1.26%, and a low-PRAL diet consumed with 8 weeks of regular aerobic exercise decreased the BMI of sedentary women by 5.17% (Table 1). These results showed that a low-PRAL diet consumed with 8 weeks of aerobic exercises was a more effective method in reducing BMI, rather than applying only aerobic exercise or a low-PRAL diet (Table 1). Massiera et al. (27) reported that Western acidic diets lead to a gradual increase in fat mass in human organisms, thus leading to the prevalence of obesity. The fact that the free-fat mass ratios did not differ significantly in our study can be considered as an indicator of the preservation of free-fat mass. However, some researchers state that physical activity, age, and protein intake, as well as long-term dietary habits up to childhood, are especially effective in preserving muscle mass (28, 29). In the literature, it is seen that longitudinal studies examining the effects of exercise and low-PRAL diet consumption on body composition and comparing exercise and different diet programs are limited (21). Contrary to our results, Jibril et al. (30) reported that there was no relationship between dietary acid load indices and body composition, despite the low-PRAL diet practices in their study on football players and referees. However, in parallel with our study, Welch et al. (29) found a positive correlation between a more low-PRAL diet and free-fat mass (%) in women aged between 18 and 79 years. They also reported a small but significant positive relationship between a more low-PRAL diet and muscle mass indices in healthy women (29). Another study reported that a low-PRAL diet may cause an increase in free-fat mass (28). Dietary factors affect metabolic acidity and alkalinity, which in turn influences metabolic outcomes such as insulin sensitivity and adiposity. High-PRAL causes metabolic acidosis, while low-PRAL diets cause a state of alkalinity. Evidence suggests that changes in pH can result in changes in body weight, body composition, and insulin sensitivity (5). People with increased diet consumption from vegetable protein tend to have a lower BMI and have been hypothesized to be less likely to be overweight (31). It is also known that the combination of taking an alkaline-based diet and physical exercise has a better effect on weight loss. The reason for this can be said to be an effective management method in reducing body weight, by exercising and eating more alkaline foods (9). The results of our research have shown similar to the previous literature, and it can be said that the low-PRAL diet consumed with an aerobic exercise program is effective in reducing BMI, fat mass ratio, and increasing free-fat mass.

When the effect of a low-PRAL diet consumed with aerobic exercises on the aerobic performance of sedentary women was examined, there was no increase in the control group, while there was an improvement in both VO_2Max_ and aerobic test duration in the exercise + Low-PRAL group, exercise group, and low-PRAL groups (Table 2). Even the highest improvement among the groups was in the exercise + low-PRAL group (VO_2Max_ = 41% and Aerobic Test Duration = 37.37%). Diet type and exercise strongly influence the body’s acid–base balance through their own mechanisms, and they can also interact that affect exercise performance. It has been hypothesized that creating a more alkaline systemic environment by reducing dietary acid load may increase the clearance of protons and inhibitory molecules affecting working muscles during exercise-induced acidosis, thereby improving aerobic and anaerobic exercise performance (13). There are also several studies examining the positive effects of supplementation with ergogenic aids such as alkaline foods with high pH, sodium bicarbonate (NaHC0_3_), or dietary nitrate for alleviating exercise performance (20, 32–36). The ingestion of NaHC0_3_ increases the concentration in the extracellular fluids HCO_3_^−^, resulting in enhanced buffering of H^+^ ions (16, 34). Increased buffering capacity from HCO_3_^−^ has been reported to improve the performance of high-intensity exercise at oxygen capacities (37–39). When the literature is reviewed, it has been described that there is an improvement in anaerobic and aerobic exercise performances (9, 40) and an alkalizing effect on blood and urine pH after a low-PRAL diet for sportive performance tests lasting 60 s to 2 min or more (41). Caciano et al. (9) found that the exhaustion times on short-term high-intensity treadmills in athletes with negative PRAL values were 21% higher than in athletes with positive PRAL values, and this is an indicator of increased anaerobic exercise performance. Despite this conclusion, Applegate et al. (13) concluded in a recent review that alkalizing diets do not have the same effects on buffering capacity and athletic performance as alkalizing agents such as NaHC0_3_. Limmer et al. (42) examined the effect of low-PRAL and high-PRAL diets on anaerobic performance in an acute experimental study conducted on 15 healthy, non-specifically trained adult volunteers, and they reported that the low-PRAL diet did not show any improvement in anaerobic performance. It seems likely that the increased anaerobic exercise performance was due to an alkalinization produced by the consumption of low-PRAL foods and possibly an increase in the availability of NaHC0_3_ (9). Because it is generally accepted that NaHC0_3_ loading also improves athletic performance by increasing the acid buffering capacity (15, 43).

In the current study, HDL-C, LDL-C, and TG levels were examined to determine the lipid profiles of sedentary women. As a result of the examinations, it was determined that there was no statistically significant difference between the groups in terms of HDL-C levels of sedentary women (*p* > 0.05), but there was a difference between TG and LDL-C levels (*p* < 0.05; Table 3). According to these results, a low-PRAL diet consumed with 8 weeks of regular exercise decreased the TG level and LDL-C level by 37.61 and 20.24%, respectively. There are studies on different diet types and exercise models in the literature (13, 44). However, longitudinal studies examining the effects of a low-PRAL diet and aerobic exercise programs on lipid profiles are limited (21). Although the mechanisms underlying the effects of exercise and nutrition on the lipid profile are unclear, it has been reported that exercise and nutrition increase the ability of skeletal muscles to use lipids instead of glycogen, thereby reducing plasma lipid levels (45). Fahlman et al. (19) reported that aerobic exercises combined with a diet for 10 weeks caused a 20% increase in HDL-C levels and a 14% decrease in TG levels. These changes in HDL-C and TG levels were achieved without any changes in body weight or dietary intake. Changes in TG and LDL-C levels are less frequently observed as a result of endurance training. Kelley et al. (46) found in their study on 788 men and women that exercise only lowered TGs, while the combination of diet and exercise + diet lowered LDL-C more than exercise alone. They observed that only in women, the LDL-C level decreased by 3% and the TG level by 5%. It shows that the consumption of a low-carbohydrate diet with exercise is also valuable in reducing the increase in TGs, as it accelerates the metabolism of fat burning in both hunger and satiety (47, 48). The pH balance in the body through low-PRAL diets is said to help reduce cholesterol levels and TGs by weakening metabolism and excretion of TGs from adipose tissue, and consumption of more alkaline minerals significantly increases fat loss (10).

## 5 Conclusion

In sum, although both aerobic exercises and a low-PRAL diet were effective on BMI, aerobic performance, and TG and LDL, it was determined that a low-PRAL diet consumed with aerobic exercises provided more effective improvements. In line with these results, to increase the quality of life, attention should be paid to the choice of alkali-based diets instead of Western diets with high acid density in the choice of diet to be consumed with regular physical activities. Thus, we can help regulate the pH level in the blood, regulate blood pressure, prevent high cholesterol, and protect against heart diseases. More longitudinal studies, including different diet types and exercise programs, are needed to generalize the effects of diets with increased alkaline densities and foods with lower acid densities on physical and physiological parameters. One of the most important limitations of this study is that the participants’ meals could not be designed by the researchers, and therefore this research could not be conducted as a randomized controlled study. Because most sedentary women are housewives and have to design their household’s meals according to their own menus. Another limitation was the low number of participants. Especially in studies on diet practices, applying diets with similar PRAL values with a higher number of participants will further increase the reliability of the results.

## Data availability statement

The raw data supporting the conclusions of this article will be made available by the authors, without undue reservation.

## Ethics statement

The studies involving humans were approved by Ethical approval of this research was obtained from Sakarya University of Applied Sciences (Protocol No: 26428519/044/14) and the research was conducted in accordance with the Declaration of Helsinki. The studies were conducted in accordance with the local legislation and institutional requirements. The participants provided their written informed consent to participate in this study.

## Author contributions

NY: Writing – original draft, Investigation, Methodology. OI: Investigation, Methodology, Writing – original draft, Conceptualization. MB: Conceptualization, Methodology, Writing – original draft. DE: Writing – original draft. GC: Conceptualization, Investigation, Methodology, Writing – original draft. DN: Funding acquisition, Supervision, Validation, Writing – original draft, Writing – review and editing.
